# BRD7 expression and c-Myc activation forms a double-negative feedback loop that controls the cell proliferation and tumor growth of nasopharyngeal carcinoma by targeting oncogenic miR-141

**DOI:** 10.1186/s13046-018-0734-2

**Published:** 2018-03-20

**Authors:** Yukun Liu, Ran Zhao, Yanmei Wei, Mengna Li, Heran Wang, Weihong Niu, Yao Zhou, Yuanzheng Qiu, Songqing Fan, Yihao Zhan, Wei Xiong, Yanhong Zhou, Xiaoling Li, Zheng Li, Guiyuan Li, Ming Zhou

**Affiliations:** 1 Hunan Cancer Hospital and The Affiliated Tumor Hospital of Xiangya School of Medicine, Central South University, Changsha, Hunan 410013 People’s Republic of China; 2 Key Laboratory of Carcinogenesis of Ministry of Health and Key Laboratory of Carcinogenesis and Cancer Invasion of Ministry of Education, The Xiangya Hospital, Central South University, Changsha, Hunan 410008 People’s Republic of China; 3 Cancer Research Institute, Central South University, Changsha, Hunan 410078 People’s Republic of China; 4 Department of Otolaryngology Head and Neck Surgery, The Xiangya Hospital, Central South University, Changsha, Hunan 410008 People’s Republic of China; 5 Department of Pathology, The Second Xiangya Hospital, Central South University, Changsha, Hunan 410011 People’s Republic of China; 6 Changzhi Medical College, Changzhi, Shanxi 046000 People’s Republic of China

**Keywords:** Nasopharyngeal carcinoma, BRD7, C-Myc, Feedback loop, miR-141, AKT pathway

## Abstract

**Background:**

miR-141 is up-regulated and plays crucial roles in nasopharyngeal carcinoma (NPC). However, the molecular mechanism underlying the dysregulation of miR-141 is still obscure.

**Methods:**

Thus, the ChIP-PCR was performed to identify the c-Myc-binding sites in miR-141 and BRD7. qRT-PCR, western blot and immunohistochemistry assays were used to detect the expression of miR-141 and its up/down stream molecules. The rescue experiments on the c-Myc/miR-141 axis were performed in vitro and in vivo.

**Results:**

Our results showed that the levels of mature miR-141, pre-miR-141 and pri-miR-141 were downregulated in c-Myc knockdown NPC cells. Meanwhile, c-Myc transactivates the expression of miR-141 by binding its promoter region. Moreover, BRD7 was identified as a co-factor of c-Myc to negatively regulate the activation of c-Myc/miR-141 axis, as well as a direct target of c-Myc. Moreover, restoration of miR-141 in c-Myc knockdown NPC cells notably rescued the effect of c-Myc on cell proliferation and tumor growth, as well as the blocking of PTEN/AKT pathway. Additionally, the expression of c-Myc was positively correlated with that of miR-141 and the clinical stages of NPC patients and negatively associated with the expression of BRD7. Our findings demonstrated that BRD7 expression and c-Myc activation forms a negative feedback loop to control the cell proliferation and tumor growth by targeting miR-141.

**Conclusions:**

These observations provide new mechanistic insights into the dysregulation of miR-141 expression and a promising therapeutic option for NPC.

**Electronic supplementary material:**

The online version of this article (10.1186/s13046-018-0734-2) contains supplementary material, which is available to authorized users.

## Background

Nasopharyngeal carcinoma (NPC) is a leading form of head and neck cancer, especially in South China. It has high metastatic potential that contributes to a high rate of local invasion and early metastasis [1]. Thus, there is a clear need to identify some sensitive biomarkers and novel therapeutic targets for diagnosis and treatment options of NPC in a premalignant stage.

Recently, non-coding RNAs have been demonstrated to represent most of the human transcriptome and are frequently dysregulated in disease pathogenesis, including cancer [2]. MicroRNAs (miRNAs) are a class of functional, short non-coding RNAs that are associated with all hallmarks in cancer initiation, progression and metastasis [3, 4]. Therefore, considering their tissue-specific profiles and stability, miRNA-based strategies are considered as promising options for early detection, accurate diagnosis and the prediction of responses to treatment in malignancies. miR-141 is a member of the miR-200 family, and it has been shown to be dysregulated in a wide variety of cancers [5, 6]. However, the mechanisms underlying the dysregulation of miR-141 are only beginning to be understood in cancer.

c-Myc is a transcription factor that generally heterodimerizes with other basic-helix–loop–helix (bHLH) proteins at thousands of genomic loci [7], which affects more than 15% of the human transcriptome [8]. Hence, c-Myc plays vital roles in almost every aspect of basic cellular processes. Aberrant activation of c-Myc is known to be a crucial hallmark of numerous cancers [9] and often leads to widespread dysregulation of miRNAs [10]. In our previous study, we found that knockdown of c-Myc significantly inhibited cell proliferation and tumor growth of NPC [11] and downregulated the level of miR-141 in NPC cells [12]. BRD7, a member of the bromodomain-containing protein family, was identified as a critical tumor suppressor in multiple types of cancers, including NPC and breast cancer [13–15] and also involved in many physiological processes [16–18]. Growing evidence has revealed that BRD7, as a ubiquitously expressed nucleic protein [17, 19], is generally involved in the transcription of specific target genes through a protein-protein interaction (PPI) with other transcription factors [20–22]. Moreover, BRD7 could negatively regulate the transcription of miR-141 in an indirect manner in NPC [23]. However, the detail mechanism underlying BRD7 and c-Myc involvement in the regulation of miR-141 is still obscure and remains to be further investigated.

In this study, we estimated the expression of BRD7 and miR-141 in c-Myc knockdown NPC cells and explored the mechanism of BRD7 and c-Myc in the transcription of miR-141. Furthermore, we performed rescue experiments to identify the functional role and molecular mechanism of the c-Myc/miR-141 axis in NPC in vitro and in vivo. Additionally, we investigated the clinical significance of c-Myc and the association of c-Myc expression with BRD7 and miR-141 in NPC patients. In summary, our findings demonstrated that BRD7 expression and c-Myc activation forms a negative feedback loop that regulates miR-141 transcription and controls cell growth and proliferation in NPC. These observations provide new mechanistic insights into the dysregulation of miR-141 expression and a promising therapeutic option for NPC in early stages.

## Methods

### Cell lines and tissue samples

The human NPC cell line 5-8F was kindly provided by the Cancer Center of Sun Yet-Sen University (Guangzhou, China). The HNE1 cell line was purchased from the cell center of the Xiangya School of Medicine (Central South University, Changsha, China). Both cell lines were preserved in our laboratory and cultured in RPMI-1640 (Hyclone, Logan, UT, USA) supplemented with 10% fetal bovine serum (FBS) (Gibco-BRL, Invitrogen, Paisley, UK) in a humidified incubator with 5% CO_2_ at 37 °C. The embryonic kidney cell line HEK293 were purchased from the American Type Culture Collection (ATCC, Manassas, VA, USA) and maintained in Dulbecco’s modified Eagle’s medium (DMEM) (Hyclone) with 10% FBS (Gibco) at 37 °C in a humidified atmosphere with 5% CO_2_.

NPC samples (*n* = 41) and non-cancerous nasopharyngeal tissues (*n* = 27) from healthy donors were collected at the Second Xiangya Hospital of Central South University (Changsha, China). The non-cancerous nasopharyngeal tissues were collected from independent patients with chronic inflammation of nasopharyngeal mucosa or polyps. The profile of clinicopathological characteristics of the NPC patients is shown in Table 1. All tissue samples were immediately snap-frozen in liquid nitrogen and stored in a freezer at − 80 °C. Tissues for immunohistochemistry were fixed in 4% paraformaldehyde and then paraffin embedded. Clinicopathological data were reviewed, and TNM staging classification was based on the criteria of the American Joint Committee on Cancer (AJCC, 6th edition).

**Table 1:** Association between the expression of c-Myc, BRD7, miR-141 and NPC clinical pathological features (*N* = 41)

	c-Myc			BRD7			miR-141		
Characteristics (N)	H (%)	L (%)	*P*	H (%)	L (%)	*P*	H (%)	L (%)	*P*
Age (year)									
≤ 40 (*n* = 7)	3(42.8)	4(57.2)	0.6786	1(14.2)	6(85.8)	0.4942	4(57.1)	3(42.9)	0.7052
> 40 (*n* = 34)	20(58.8)	14(41.2)		9(26.4)	25(73.6)		22(64.7)	12(35.3)	
Gender									
Female (*n* = 9)	6(66.6)	3(33.4)	0.7061	2(22.2)	7(77.8)	0.8639	8(88.8)	1(11.2)	0.0725
Male (*n* = 32)	17(53.1)	15(46.9)		8(47.1)	24(52.9)		18(56.2)	14(43.8)	
Histological type									
DNC (*n* = 20)	12(60.0)	8(40.0)	0.7557	5(25.0)	15(75.0)	0.9293	14(70.0)	6(30.0)	0.3929
UDNC (*n* = 21)	11(52.3)	10(47.7)		5(23.8)	16(76.2)		12(57.1)	9(42.9)	
Clinical stage									
Stage I (*n* = 7)	1(14.2)	6(85.8)	0.0140	4(57.1)	3(42.9)	0.0475	2(28.6)	5(71.4)	0.0246
Stage II (*n* = 19)	10(52.6)	9(47.4)		2(10.5)	17(89.5)		11(57.8)	8(42.2)	
Stage III-IV (*n* = 15)	12(80.0)	3(20.0)		4(26.6)	11(73.4)		13(86.6)	2(13.4)	

*Abbreviations*: DNC, differentiated non-keratinized nasopharyngeal carcinoma; UDNC, undifferentiated non-keratinized nasopharyngeal carcinoma; H, high expression; L, low expression

### Plasmids, oligonucleotides and transfections

The interfering plasmid (pRNAT-U6.1/shc-Myc) expressing short hairpin RNA (shRNA) for c-Myc knockdown, the expression plasmid encoding the full-length open reading frame of human c-Myc with 2Flag tags (pIRES2-EGFP/2Flag-c-Myc), and the plasmid encoding the full-length open reading frame of human BRD7 with 3Flag tags (pIRESneo3/3Flag-BRD7) or GFP tag (pEGFP-C2/BRD7) were constructed by our laboratory. The hsa-miR-141 mimic (miR-141) and negative control (miR-NC) were purchased from RiBoBio (Guangdong, China).

Cells were transfected with plasmids and/or oligonucleotides using Lipofectamine3000 Reagent (Invitrogen, Carlsbad, CA, USA) according to the manufacturer’s protocol. Briefly, cells in logarithmic growth phase were trypsinized, counted and seeded in a 6-well plate to ensure 60–80% cell confluence on the next day for transfection. Transfection of cells with oligonucleotides was performed at a final concentration of 50 nM.

The c-Myc knockdown stable cell lines (5-8F/shc-Myc and HNE1/shc-Myc) and the control cell lines (5-8F/shCtrl and HNE1/shCtrl) were transfected with pRNAT-U6.1/shc-Myc and pRNAT-U6.1/shCtrl, respectively, and then screened under G418 (Invitrogen). Finally, stable pool clones were obtained.

### qRT-PCR

Total RNA was isolated from tissue samples and cell lines using TRIzol reagent (Invitrogen) according to the manufacturer’s protocol. The levels of mature miR-141 (miR-141) and precursor miR-141 (pre-miR-141) were evaluated using the miDETECT A Track miRNA qRT-PCR Kit (RiBoBio). The U6 small nuclear RNA (RNU6B) (RiBoBio) was used for normalization. The expression of primary miR-141 transcript (pri-miR-141), c-Myc and BRD7 was measured by qRT-PCR according to the instructions of the SYBR Premix Ex Taq (TaKaRa, Dalian, China). The GAPDH mRNA level was used for normalization. The relative expression ratio was calculated using the 2^−ΔΔCT^ method. The primers for miR-141, pre-miR-141, U6, pri-miR-141, c-Myc, BRD7, and GAPDH were described previously [23]. PCRs of each sample were conducted at least in triplicate.

### Western blot analysis

Cells and tissues were lysed in RIPA buffer in the presence of Protease Inhibitor Cocktail and PhoSTOP (Roche, Basel, Switzerland). Protein was quantified using a BCA Protein Assay Kit (Pierce Biotechnology, Rockford, IL, USA). Protein (30–80 μg) was separated by 8–12% sodium dodecyl sulfate polyacrylamide gel electrophoresis, and transferred onto polyvinylidene fluoride (PVDF) membranes (Millipore, Billerica, MA, USA). The membranes were blocked with 5% non-fat milk in Tris-buffered saline and then incubated with primary antibodies at 4 °C overnight. The primary antibodies used were anti-c-Myc (dilution 1:1000; CST, Danvers, MA, USA), anti-Dicer (dilution 1:1000; CST), anti-Drosha (dilution 1:1000; CST), anti-BRD7 (dilution 1:500; ProteinTech, Wuhan, China), anti-Flag (dilution 1:2000; Sigma, St. Louis, MO, USA), anti-GFP (dilution 1:2000; ProteinTech), anti-β-actin (dilution 1:1000; Santa Cruz, CA, USA), anti-PARP (dilution 1:1000; CST), anti-cleaved-PRAP (C-PARP) (dilution 1:1000; CST), anti-CCND1 (dilution 1:500; Santa Cruz), anti-CDK4 (dilution 1:500; Santa Cruz), anti-PTEN (dilution 1:500; Bioworld Technology, Atlanta, GA, USA), anti-pS473AKT (dilution 1:500; Bioworld Technology), anti-AKT (dilution 1:500; Bioworld Technology), anti-p27 (dilution 1:1000; CST), anti-Caspase9 (dilution 1:500; Santa Cruz) and anti-cleaved-Caspase9 (dilution 1:500; Santa Cruz). Membranes were then washed three times in TBST solution for 10 min each time and incubated with secondary antibodies. Signals were detected with an enhanced chemiluminescence detection system (Bio-Rad, Hercules, CA, USA).

### The luciferase reporter assays

The recombinant reporter vectors of the miR-141 promoter (pGL3/miR-141P) and BRD7 promoter (pGL3/BRD7P) were constructed by our laboratory as described previously [23, 24]. The cells were seeded in 24-well plates. After 24 h, the cells were transfected with recombinant reporter vector or pGL3-Enhancer empty vector (Promega, Madison, WI, USA), together with the pRL-TK vector (Promega) containing the Renilla luciferase gene. Transfection was performed using Lipofectamine 3000 (Invitrogen). Cells were harvested at 36 h post-transfection. Firefly and Renilla luciferase activities were measured using a Dual-luciferase reporter kit (Promega) according to the manufacturer’s protocol. Firefly luciferase activity was normalized to Renilla luciferase activity.

### Chromatin immunoprecipitation (ChIP) assays

HEK293 cells transfected with pIRES2-EGFP/2Flag-c-Myc plasmid were cross-linked in 1% formaldehyde for 10 min at 37 °C. DNA from fixed chromatin cells was then subjected to immunoprecipitation using a ChIP assay kit (Millipore) and antibodies against Flag tags or mouse IgG according to the manufacturer’s protocol. The purified DNA was used for the following PCRs. The primers for confirmation of the c-Myc-binding sites in the miR-141 and BRD7 promoter in PCR reactions are described in Additional file 1: Table S1.

### Immunofluorescence (IF) and co-immunoprecipitation (co-IP)

Subcellular localization of endogenous c-Myc and exogenous BRD7 was analyzed by IF. Briefly, 5-8F and HNE1 cells were seeded on glass coverslips, transfected with pEGFP-C2/BRD7, fixed with 4% paraformaldehyde for 15 min at room temperature and washed with TBS. Cells were then permeabilized with 0.3% Triton X-100 for 5 min at room temperature. Cells were washed 3 times with TBS and incubated for 15 min in 5% goat serum and then incubated at 4 °C overnight with the primary antibody anti-c-Myc (dilution 1:800; CST). After three 5-min washes with TBS, cells were incubated for 1 h at 37 °C in the dark with the secondary antibody Alexa Fluor 568 Donkey anti-Rabbit IgG (H + L) (dilution 1:1000; Invitrogen). Coverslips were washed with TBS and visualized using an epifluorescence microscope.

For Co-IP, HEK293 cells transfected with pIRES2-EGFP/2Flag-c-Myc plasmid and pEGFP-BRD7 plasmid together were lysed with cell lysis buffer for western blotting and IP (Beyotime, Beijing, China). They were then pre-mixed with protease inhibitor cocktail (Roche) on ice for 20 min and centrifuged at 12,000 g for 15 min. The supernatants were incubated at 4 °C overnight with 30 μl of protein A/G magnetic beads (Biomake, Houston, TX, USA) pre-coated with anti-Flag antibodies (Sigma) or anti-mouse IgG (Santa Cruz). The immunocomplexes were isolated and purified according to the manufacturer’s protocol, and then were subjected to western blot analysis.

### Cell proliferation (MTS) and colony-forming assays

The cell proliferation capacity was estimated with the MTS kit (Biomake) following the manufacturer’s protocol. Briefly, cells were seeded at a density of 1 × 10^3^ cells/well in a 96-well plate and cultured for 48 or 96 h. Ten microliters of MTS solution was added to each well, and the samples were incubated at 37 °C for 3 h before the absorbance was measured at 450 nm.

For colony-forming assays, approximately 150 cells per well were added to a 6-well plate, with three wells per sample. After a 14-day incubation, the cells were washed twice with PBS and stained with Giemsa solution (Beyotime, Beijing, China). The plate colony-forming efficiency was calculated as (number of colonies/number of cells inoculated) × 100%. All experiments were performed at least in triplicate.

### Flow cytometry for cell cycle and apoptosis analysis

For cell cycle analysis, cells were harvested and fixed in 70% ethanol for 24 h at − 20 °C. The cells were then treated with RNase A and stained with 25 μg/ml propidium iodide (PI). For cell apoptosis analysis, cells were treated with serum starvation for 24 h, and then the ratio of apoptotic cells was determined using an Annexin V-PE/7-AAD double staining kit (BD Biosciences, MD, USA). Samples were all analyzed using a MoFlo™ XDP High-Performance Cell Sorter (Beckman Coulter, CA, USA), and the data were analyzed using Summit v.5.2 software according to the manufacturer’s protocol. At least three independent experiments were performed.

### Immunohistochemical (IHC) staining, in situ hybridization (ISH) and scores

For IHC staining, the sections were incubated with anti-c-Myc (dilution 1:100; Millipore), anti-PTEN (dilution 1:100; Bioworld), anti-pS473AKT (dilution 1:100; Bioworld), anti-p27 (dilution 1:100; CST), and anti-CCND1 (dilution 1:100; Santa Cruz) antibodies. The immune complex was visualized by the MaxVision HRP-polymer IHC Kit Detection System, Peroxidase/DAB, Rabbit/Mouse (MaxVision, Fuzhou, China) according to the manufacturer’s protocol. The nuclei were counterstained with hematoxylin (Beyotime). For ISH staining, the mature Hsa-miR-141-specific probe and negative control (Scramble) were purchased from Sangon (Sangon Biotech, Shanghai, China). An in situ hybridization kit (Boster, Wuhan, China) was used for hybridization of the probe according to the manufacturer’s instructions.

Scores for IHC and ISH analysis. IHC and ISH staining were independently evaluated at 200× magnification using light microscopy by two pathologists who were blinded to the clinicopathological data. A semiquantitative evaluation of c-Myc, BRD7 protein and mature miR-141 was performed using a method described in our previous work [23].

### Tumor growth xenograft model

A total volume of 200 μl of 5-8F/shc-Myc and 5-8F/shCtrl cells (1× 10^7^ cells) transfected with miR-141 mimic (miR-141) or negative control (miR-NC) was inoculated subcutaneously into the left flanks of 6-week-old female nude mice. Mice were checked every 4 days. Tumor volume was evaluated using the following formula: volume = (width + length)/2 × width × length × 0.5236. All three groups were killed after 28 days. All tumor grafts were excised, weighed, harvested, fixed and embedded. The anti-Ki67 antibody (dilution 1:100, Bioworld) was used to detect the proliferation marker Ki67 using IHC procedures, and a TUNEL assay was used to detect apoptosis in situ with paraffin-embedded xenograft sections using the DeadEnd Colorimetric TUNEL Detection Kit (Promega) according to the manufacturer’s protocols. Samples were observed using an Olympus microscope (Olympus, Tokyo, Japan). The proliferative and apoptosis index scores were measured as the mean percentage of nuclei that stained positive for Ki67 and TUNEL cells in 10 different 200× fields.

### Statistical analysis

The relationships between the expression of c-Myc, BRD7, miR-141 and clinicopathological characteristics in NPC were tested using a chi-squared test. The Spearman’s rank correlation coefficient was used to assess the significance of the association among c-Myc, BRD7 and miR-141 expression in NPC patients. The differences between the groups were analyzed using a Student’s t-test when there were only two groups or using a one-way ANOVA when there were more than two groups. All statistical analyses were performed using SPSS software (SPSS, Chicago, IL, USA). A two-tailed value of *P* < 0.05 was considered statistically significant.

## Results

### C-Myc transactivates miR-141 expression

**Fig. 1:**
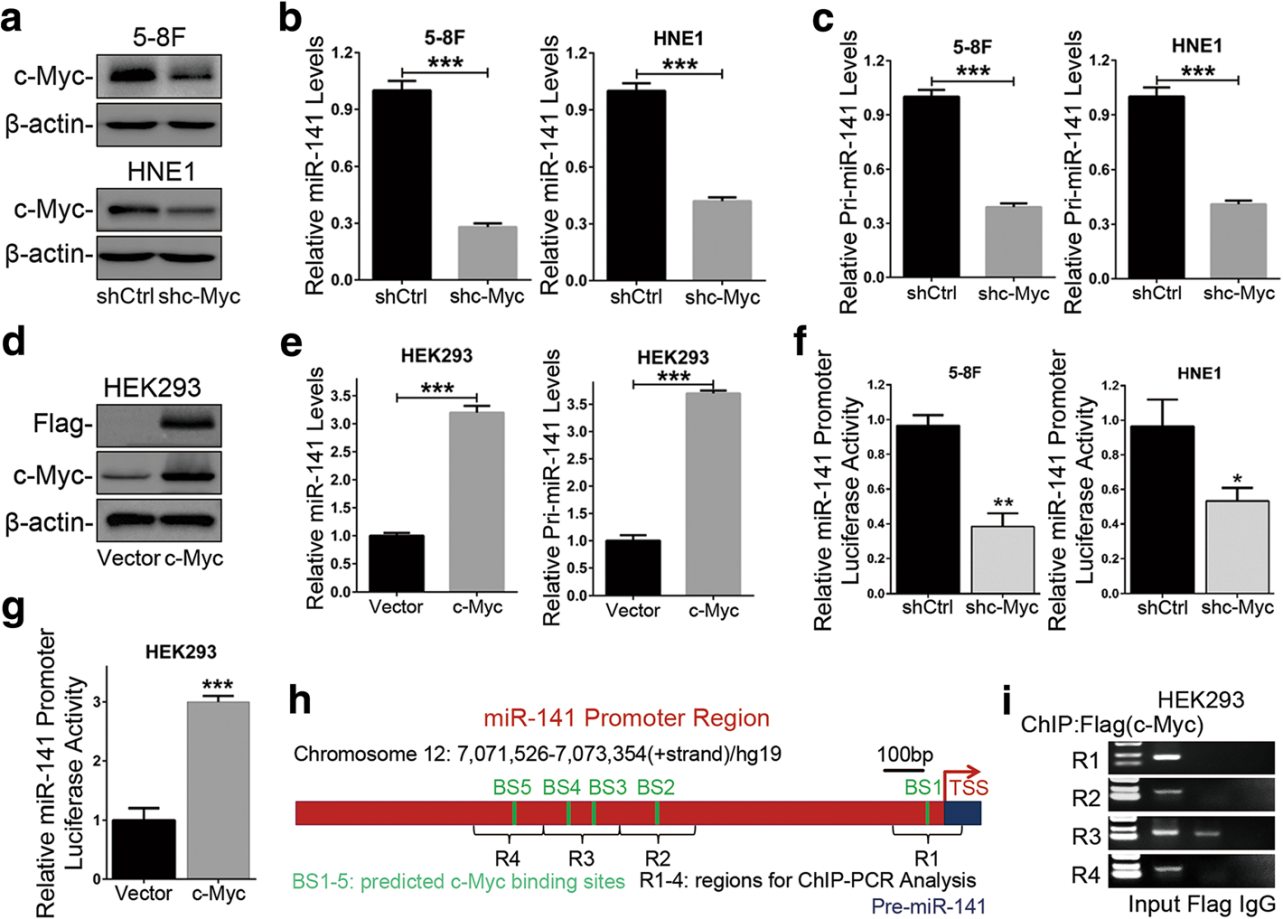
c-Myc promoted the transcription of miR-141 by directly binding its promoter. **a** Western blotting confirmed the expression of endogenous c-Myc in c-Myc knockdown stable 5-8F and HNE1 cells (shc-Myc) and control cells (shCtrl). **b** and **c** The levels of miR-141 and pri-miR-141 were assessed by qRT-PCR in c-Myc knockdown 5-8F and HNE1 cells. **d** Western blotting validated the overexpression of c-Myc in c-Myc-overexpressing HEK293 cells (c-Myc) and control cells (Vector). **e** The levels of miR-141 and pri-miR-141 were analyzed by qRT-PCR in c-Myc-overexpressing HEK293 cells. **f** and **g** The dual-luciferase reporter assays detected the relative miR-141 promoter activity in c-Myc knockdown 5-8F and HNE1 cells and c-Myc-overexpressing HEK293 cells, normalized to pRL-TK. **h** A schematic diagram showing the locations of predicted c-Myc-binding sites in the miR-141 promoter region on chromosome 12p13.31 (forward strand) and the amplified regions of PCR for ChIP assays. BS1-BS5: the 5 binding sites of c-Myc in the miR-141 promoter region predicted by the JASPAR Database. **i** ChIP-PCR assays using antibodies specific for Flag tags validated the c-Myc-binding sites in the miR-141 promoter. **a** and **d** β-actin served as an internal control. **b**, **c** and **e** GAPDH served as an internal control for pri-miR-141 detection, and U6 for miR-141 detection. The error bars represent the mean ± S.E.M. *P < 0.05, **P < 0.01, ***P < 0.001

We first evaluated the level of miR-141 in c-Myc knockdown stable NPC cells (Fig. 1a). The results showed that the level of miR-141 was significantly downregulated in both c-Myc knockdown 5-8F and HNE1 cells (Fig. 1b). Considering the general principles of miRNA biosynthesis, we further examined the abundance of pri-miR-141 and pre-miR-141 and the expression of Dicer and Drosha, two key enzymes in the miRNA maturation. As a result, the levels of both pri-miR-141 and pre-miR-141 were correlated with the miR-141 level in c-Myc knockdown cells (Fig. 1c and Additional file 2: Figure S1a). Nevertheless, knocking down c-Myc had no obvious effect on the expression of Dicer and Drosha protein (Additional file 2: Figure S1b). Conversely, in HEK293 cells with endogenous low-level expression of c-Myc, overexpression of c-Myc significantly elevated the abundance of miR-141, pre-miR-141 and pri-miR-141 (Fig. 1d-e and Additional file 2: Figure S1c) but did not obviously influence the Dicer and Drosha protein expression (Additional file 2: Figure S1d).

Considering that c-Myc is a transcription factor and that its aberrant activation often leads to widespread dysregulation of miRNAs, we further detected the effect of c-Myc on the promoter activity of miR-141. The results showed that the miR-141 promoter activity was significantly reduced in both c-Myc knockdown cells (Fig. 1f). Meanwhile, the miR-141 promoter activity was significantly increased in c-Myc-overexpressing HEK293 cells (Fig. 1g). Furthermore, we mapped the c-Myc-binding profile using the JASPAR Database [25]. Within the miR-141 promoter region, 10-putative c-Myc-binding sites were present (Additional file 3: Table S2). Considering the location of the miR-141-encoding gene on chromosome 12 forward strand, we conducted ChIP-PCR assays and tested the 5-putative c-Myc-binding sites (named BS1, BS2, BS3, BS4 and BS5) (Fig. 1h). The assays validated that c-Myc binds directly to the region covering the BS3 and BS4 sites in the miR-141 promoter (Fig. 1i). Altogether, these data demonstrated that c-Myc activates miR-141 expression by directly binding to its promoter region.

### A negative feedback loop formed by BRD7 and c-Myc is involved in the regulation of miR-141 expression

**Fig. 2:**
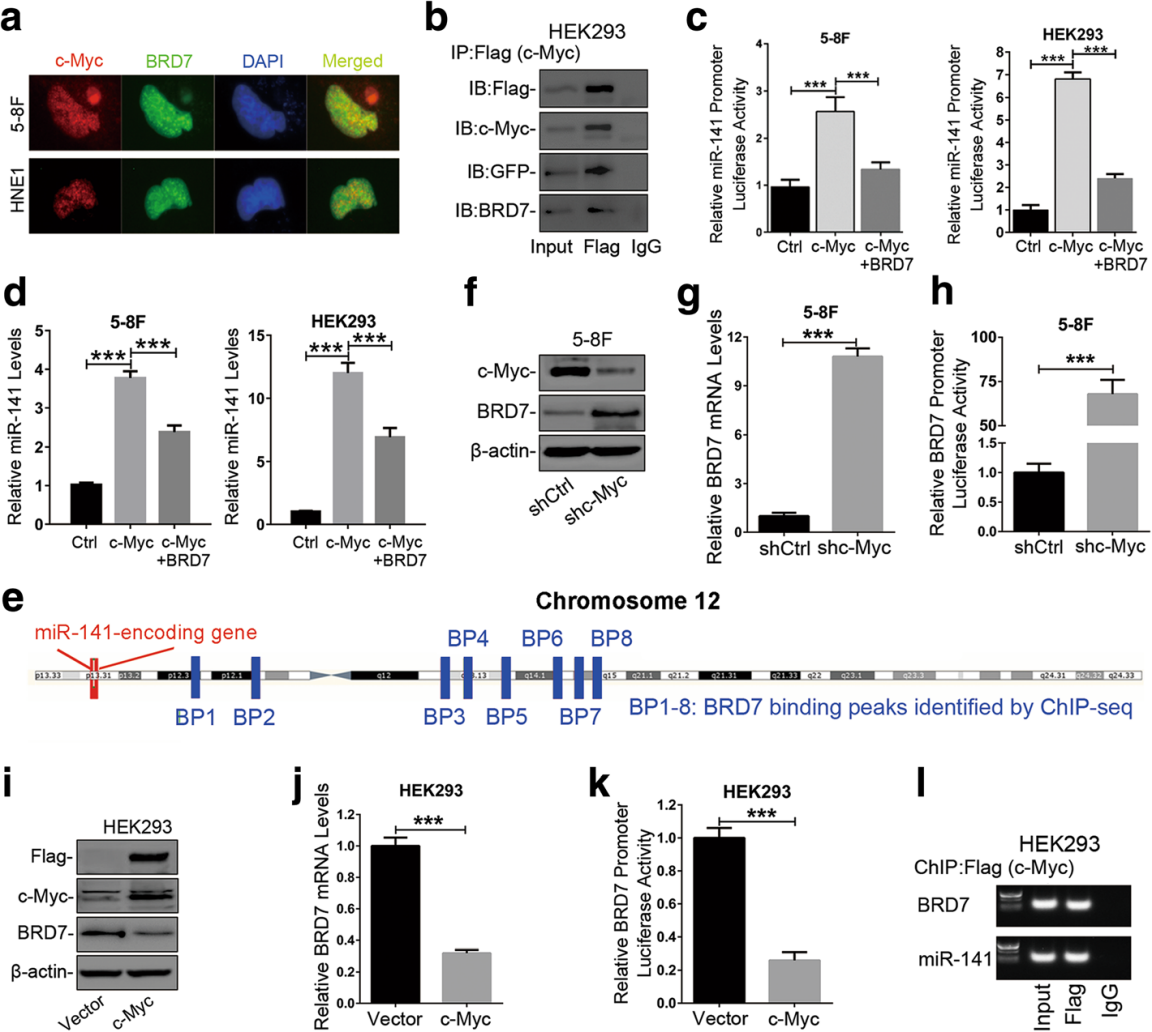
BRD7 formed a negative feedback loop with c-Myc in miR-141 transcription. **a** IF detected the subcellular distribution pattern of BRD7 and c-Myc protein, as well as the colocalization of BRD7 with endogenous c-Myc in 5-8F and HNE1 cells transfected with pEGFP-C2/BRD7. c-Myc: endogenous c-Myc protein, BRD7: EGFP-tagged BRD7 protein. **b** Co-IP assays and western blotting confirmed the interaction between BRD7 (GFP-tagged) and c-Myc (Flag-tagged) in HEK293 cells. **c** The dual-luciferase reporter assays assessed the miR-141 activity promoter when overexpressing BRD7 in c-Myc-overexpressing 5-8F and HEK293 cells (c-Myc) and control cells (Vector). **d** qRT-PCR assays evaluated the expression of miR-141 in c-Myc-overexpressing 5-8F and HEK293 cells when overexpressing BRD7. **e** A schematic diagram showing the locations of BRD7-binding peaks in chromosome 12 identified by ChIP-sequencing. **f** Western blotting and **g** qRT-PCR assays evaluated the expression of BRD7 protein and mRNA, respectively, in c-Myc knockdown 5-8F cells (shc-Myc) and control cells (shCtrl). **h** The dual-luciferase reporter assays determined the BRD7 promoter activity in c-Myc knockdown 5-8F cells and control cells. **i** Western blotting and **j** qRT-PCR assays analyzed the expression of BRD7 protein and mRNA in c-Myc-overexpressing HEK293 cells and controls. **k** The dual-luciferase reporter assays determined the BRD7 promoter activity in c-Myc-overexpressing HEK293 cells. **l** ChIP-PCR assays using antibodies specific for Flag tags validated the c-Myc-binding sites in the BRD7 promoter. miR-141 was used as a positive control in this experiment. **b**, **f** and **i** β-actin served as an internal control. D), G) and J) U6 or GAPDH served as an internal control. **c**, **d**, **g**, **h**, **j** and **k** The error bars represent the mean ± S.E.M. ***P < 0.001

c-Myc plays a dual role in the transcription, activation or repression of its target genes, and this transition practically depends on its partner protein [26]. Likewise, BRD7 often regulates specific target genes through interaction with other transcription factors [20–22]. Additionally, we have identified that BRD7, as a transcriptional regulator, could repress the transcription of miR-141 in an indirect manner [23]. Therefore, we want to know whether BRD7 could serve as a cofactor of c-Myc to regulate the expression of miR-141. Firstly, we predicted that BRD7 might be one of c-Myc interacting proteins by interrogating the annotated PPI predictions data from the Fpclass database (Additional file 4: Table S3). Then, the IF analysis showed that BRD7 was colocalized with c-Myc in a punctate distribution pattern (Fig. 2a), and the Co-IP experiments further revealed that BRD7 was co-immunoprecipitated with c-Myc (Fig. 2b). All of these data supported that BRD7 can indeed serve as an interactor of c-Myc. Furthermore, we detected the effect of BRD7 on the transcription activity and expression of miR-141 regulated by c-Myc. As a result, ectopic expression of BRD7 significantly repressed the c-Myc-induced activation of the miR-141 promoter (Fig. 2c and Additional file 2: Figure S2a) as well as the expression of miR-141 (Fig. 2d and Additional file 2: Figure S 2b). Additionally, we examined that ectopic expression of BRD7 did not affect the expression of c-Myc protein and mRNA (Additional file 2: Figure S2c and d). Importantly, we have previously performed an integrative analysis of genome-wide chromatin occupancy of BRD7 by ChIP-sequencing [27] and noted that there were 8 binding peaks of BRD7 on chromosome 12 but no more than one in the miR-141 promoter region (Fig. 2e). These observations demonstrated that BRD7 could serve as a cofactor of c-Myc to be negatively involved in the transcription of miR-141 regulated by c-Myc.

Since BRD7, as co-factor of c-Myc, is negatively involved in the transcription of miR-141, we still want to know whether c-Myc feedbackly affect the expression of BRD7. As a result, knockdown of c-Myc observably enhanced the abundance of BRD7 protein and mRNA as well as the BRD7 promoter activity in both 5-8F and HNE1 cells (Fig. 2f-h and Additional file 2: Figure S2e-g). On the contrary, overexpression of c-Myc could remarkably downregulate the expression of BRD7 protein and mRNA as well as the BRD7 promoter activity in HEK293 cells (Fig. 2i, j and k). However, overexpression of c-Myc could not change the expression and the promoter activity of BRD7 in 5-8F and HNE1 cells (Additional file 2: Figure S2 h, i and j), suggesting that the high level of endogenous c-Myc is sufficient to inactivate the transcription of BRD7 in NPC cells. Beyond that, the ChIP-PCR experiments further revealed that c-Myc could directly bind to the BRD7 promoter region (Fig. 2l). These results suggest that BRD7 expression and c-Myc activation form a negative feedback loop responsible for the regulation of miR-141 transcription.

### Restoring the expression of miR-141 significantly rescues the tumor suppressive effects of c-Myc knockdown in NPC cells

**Fig. 3:**
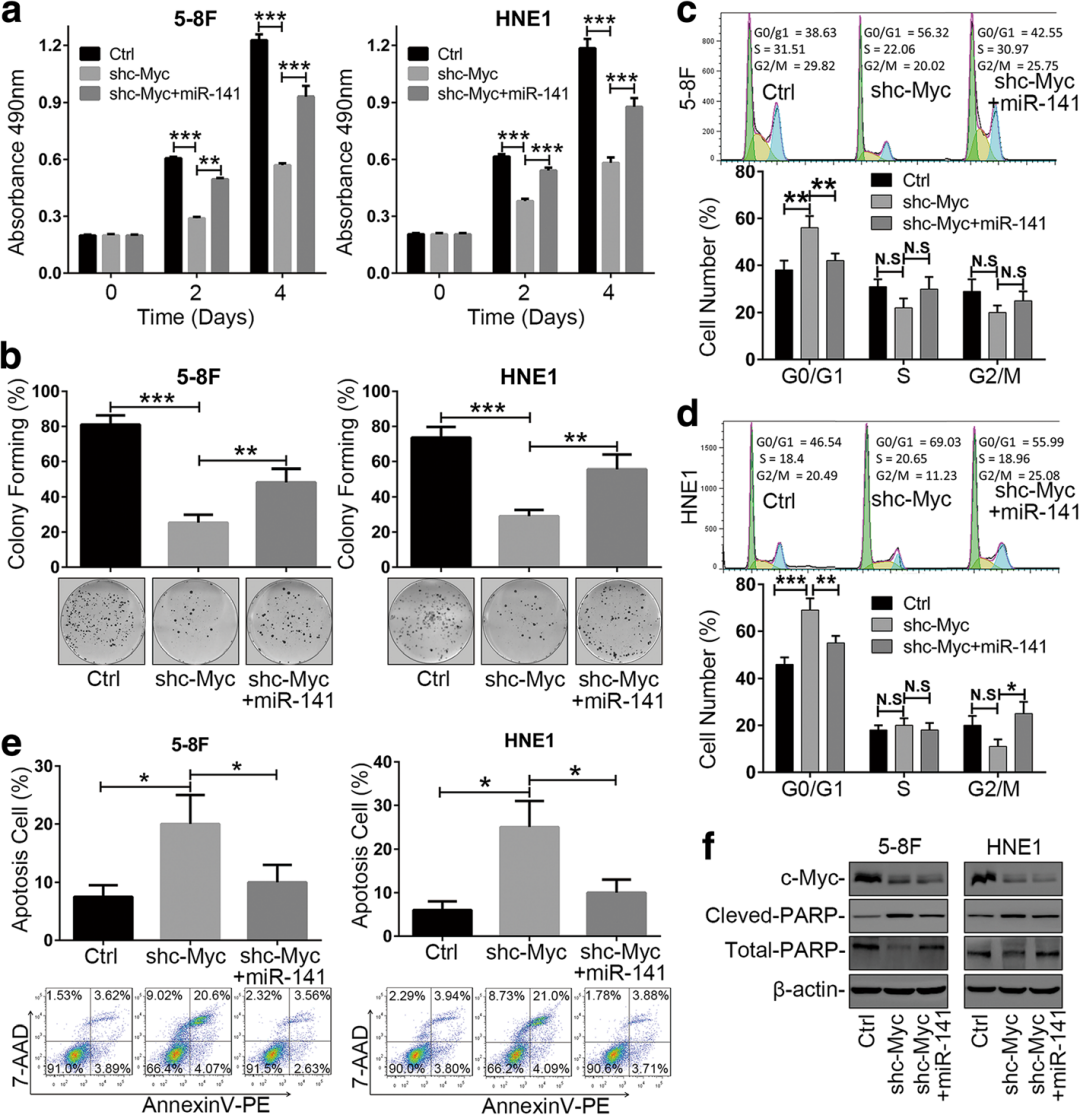
Restoring miR-141 levels rescued c-Myc knockdown-mediated cell proliferation inhibition and apoptosis promotion in NPC cells. **a** MTS assays of c-Myc knockdown NPC cells transfected with miR-141 or miR-NC. Ctrl: shCtrl+miR-NC; shc-Myc: shc-Myc + miR-NC. **b** Colony-forming assay images (lower panel) and quantification of colony number percentages (upper panel). **c** and **d** Cell cycle analysis and **e** Annexin V-PE and 7-AAD double staining analysis of cell apoptosis by flow cytometry. **f** Expression of the apoptosis marker C-PARP was determined by western blotting. β-actin served as an internal control. **a**-**e** The error bars represent the mean ± S.E.M. *P < 0.05, **P < 0.01, ***P < 0.001, N.S=No Significance

To further investigate the role of c-Myc transactivating miR-141 expression, we performed rescue experiments in c-Myc knockdown NPC cells. The level of miR-141 restoration was confirmed by qRT-PCR assays (Additional file 2: Figure S3). The MTS assays and colony-forming experiments showed that restoring the expression of miR-141 significantly reversed cell proliferation inhibition by c-Myc knockdown (Fig. 3a and b). Meanwhile, the cell cycle analysis further validated that the restoration of miR-141 levels elicited a notable reduction of the G0/G1 population compared to the c-Myc knockdown controls (Fig. 3c and d). Furthermore, restoring the expression of miR-141 significantly recovered the percentage of apoptotic cells (Fig. 3e) and the expression of cleaved-PARP (C-PARP) (Fig. 3f) compared to the c-Myc knockdown controls. These results together demonstrated that the c-Myc/miR-141 axis has a critical role in cell proliferation and apoptotic responses, suggesting that c-Myc exerted its oncogenic through transactivating miR-141 expression in NPC progression.

### miR-141 can reverse c-Myc knockdown-mediated tumor growth suppression in vivo

**Fig. 4:**
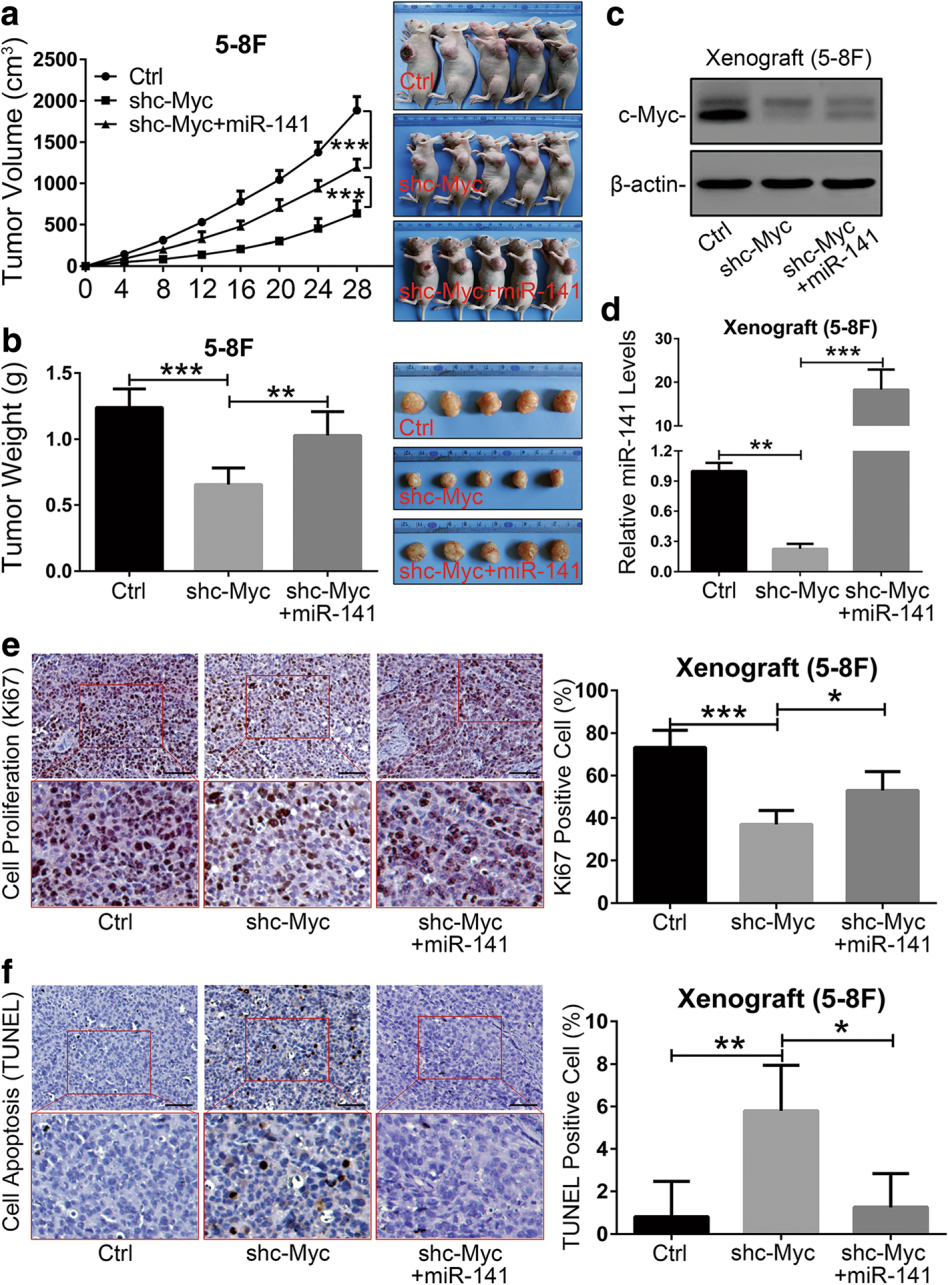
miR-141 reversed the tumor growth suppressive effect of c-Myc knockdown in vivo. **a** Tumor growth curve (left panel) and images (right panel) of the 5-8F xenograft model in nude mice. **b** Tumor weight quantification (left panel) and images (right panel) (*N* = 5). **c** Western blotting and **d** qRT-PCR assays confirmed the expression of c-Myc and miR-141 in xenografts, respectively. β-actin and U6 served as internal controls, respectively. **e** IHC (DAB staining) for quantification of the proliferation marker Ki67 (right panel) and images (left panel) and **f** TUNEL (DAB staining) for quantification of apoptosis (right panel) and images (left panel). **e** and **f** Original magnification, 200×; the scale bars represent 50 μm. **a**, **b**, **d**, **e** and **f** The error bars represent the mean ± S.E.M. *P < 0.05, **P < 0.01, ***P < 0.001

To corroborate our in vitro observations, we conducted in vivo experiments with xenograft tumor models in nude mice. As a result, the growth rate and weight of the xenograft tumors with miR-141 restoration were significantly increased compared with those of the c-Myc knockdown group (Fig. 4a and b). In addition, the expression of c-Myc and miR-141 in xenografts was confirmed by western blotting and qRT-PCR assays, respectively (Fig. 4c and d). To further validate the effect of miR-141 restoration on tumor growth in vivo, we determined the percentage of cells expressing the proliferation marker Ki67 and the percentage of TUNEL-positive cells. Consistent with our in vitro data, the restoration of miR-141 caused a significant increase in the number of Ki67^+^ cells and a reduction in TUNEL^+^ cells in c-Myc knockdown xenograft tumors when compared with the c-Myc knockdown controls (Fig. 4e and f). All of these in vivo findings corresponded with our in vitro results and supported that c-Myc, as an oncogenic transcription factor, promoted tumor growth through transactivating miR-141 expression in NPC.

### C-Myc promotes NPC progression through the miR-141/PTEN/AKT pathway

**Fig. 5:**
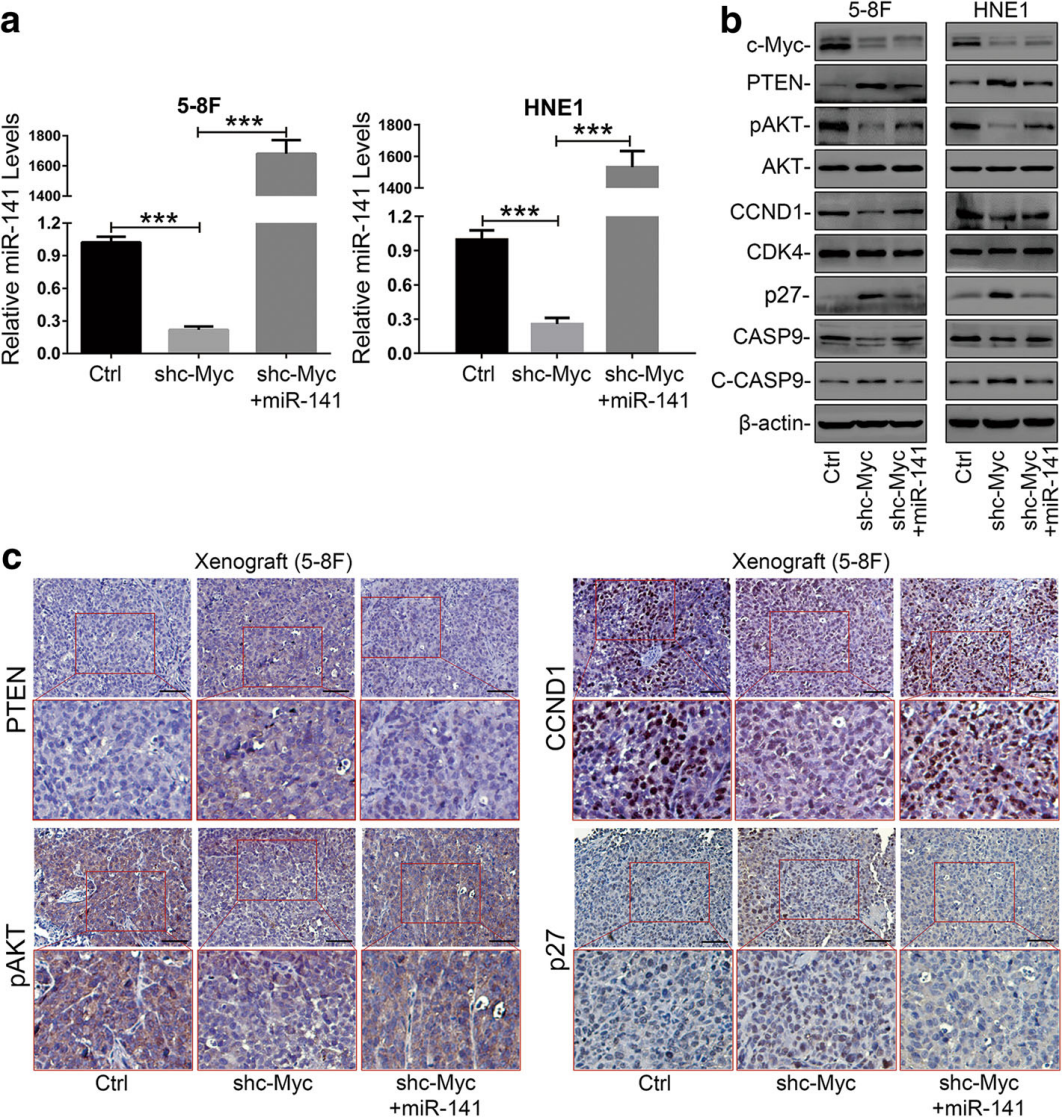
c-Myc regulated NPC growth through the miR-141/PTEN/AKT pathway. **a** qRT-PCR assays confirmed the level of miR-141 restoration in c-Myc knockdown 5-8F and HNE1 cells. U6 served as an internal control. **b** Western blotting confirmed the expression of differently expressed proteins downstream in the PTEN/AKT signaling, including p27, CCND1, CDK4, Caspase9, and Cleaved-caspase9, in miR-141-restoring 5-8F and HNE1 cells and the controls. β-actin served as an internal control. Ctrl: shCtrl+miR-NC, shc-Myc: shc-Myc + miR-NC. **c** IHC (DAB staining) for PTEN, pAKT, p27, and CCND1 in the 5-8F xenografts. Three tumors were analyzed per group using IHC staining for the molecules mentioned above. Original magnification, 200×; the scale bar represents 50 μm

We have previously demonstrated that miR-141 could directly target the 3′ untranslated region (3’UTR) of PTEN [12], and the restoration of miR-141 could significantly reverse the tumor suppressive effect of BRD7 on tumor growth through the PTEN/AKT pathway in NPC [23]. Thus, we reasoned that c-Myc can promote NPC growth through the PTEN/AKT pathway by activating miR-141 expression. First, we confirmed the restoration of miR-141 levels in c-Myc knockdown NPC cells (Fig. 5a). Then, we detected the expression of PTEN, AKT and pAKT. The immunoblot showed that knockdown of c-Myc in either 5-8F or HNE1 cells led to an increase in the expression of PTEN protein and induced a notable dephosphorylation of pAKT compared with the controls (Fig. 5b). Conversely, restoring miR-141 levels reduced the protein expression of PTEN and stimulated AKT phosphorylation in c-Myc knockdown NPC cells (Fig. 5b), suggesting that the activation of the AKT pathway by c-Myc is at least partially dependent on the repression of PTEN by miR-141 in NPC cells. Notably, IHC staining of xenografts showed that knockdown of c-Myc induced the expression of PTEN and decreased the phosphorylation level of pAKT compared with the control group (Fig. 5c). Collectively, these data illustrated that c-Myc activates AKT signals by downregulating the expression of PTEN protein at least partially via transcriptionally modulating miR-141 expression in vitro and in vivo.

To identify the effectors responsible for the phenotypes observed in vitro and in vivo, we examined alterations of the key components downstream in the PTEN/AKT pathway. The immunoblot results revealed that the restoration of miR-141 in c-Myc knockdown cells resulted in a reduction of p27 protein and an increase of CCND1, while CDK4 expression remained unaltered compared to the controls (Fig. 5b). Consistent with the in vitro findings, IHC staining of xenografts further confirmed that the expression of p27 was decreased and that CCND1 was increased in miR-141-restoring tumors when compared to the c-Myc knockdown controls (Fig. 5c). Additionally, Caspase9 cleavage was blocked when miR-141 was restored in c-Myc knockdown tumors (Fig. 5c). Collectively, these data further validated that the c-Myc/miR-141 axis controls NPC growth at least partially through modulating the PTEN/AKT pathway.

### Clinical significance of the feedback loop in miR-141 transcription in human NPC

**Fig. 6:**
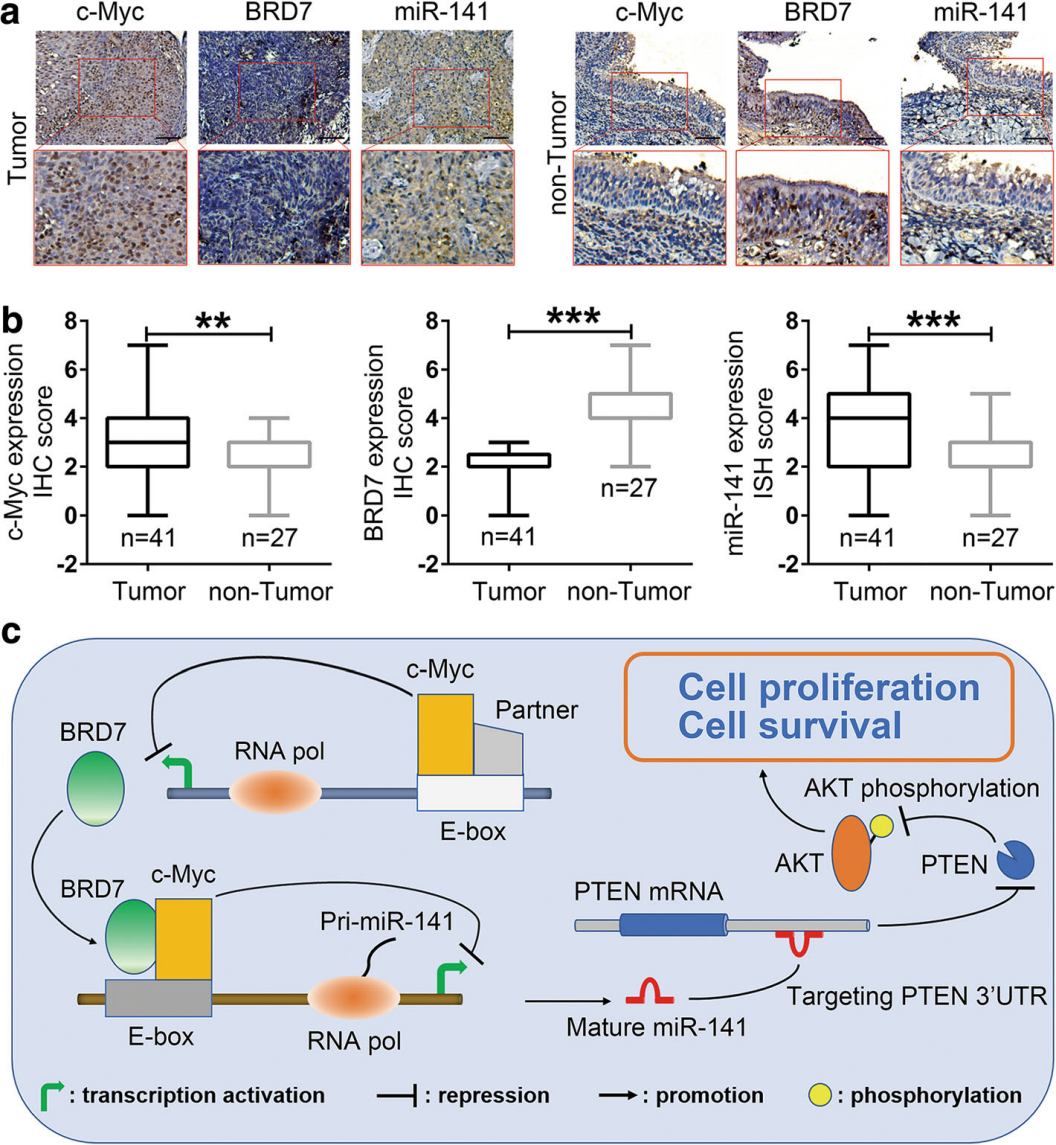
The clinical significance of c-Myc and the mechanism of BRD7 and c-Myc in NPC progression. **a** The expression of c-Myc, BRD7 and miR-141 was analyzed by IHC or ISH in NPC (Tumor, *N* = 41) and non-cancerous nasopharyngeal controls (non-Tumor, *N* = 27), respectively. Original magnification, 200×; the scale bars represent 50 μm. **b** Box diagram of c-Myc, BRD7 and miR-141 expression in NPC and non-cancerous nasopharyngeal control tissues. The whiskers represent the minimum and maximum values for each group. **P < 0.01, ***P < 0.001. **c** A schematic map showing the mechanism of the negative feedback loop between BRD7 and c-Myc in miR-141 transcription that contributes to NPC cell proliferation and survival. In this model, c-Myc binds directly to the BRD7 promoter and represses its transcription; meanwhile, BRD7 interacts with c-Myc and functions as a cofactor of c-Myc in the transcription of miR-141, which induces the inhibition of miR-141 transcription; miR-141 activates the AKT pathway through directly targeting PTEN and then promotes the cell proliferation and survival of NPC

To assess the significance of the feedback loop between BRD7 and c-Myc in NPC progression, we detected their expression in NPC tissues and non-cancerous NP samples (Table 1, Fig. 6a). As a result, most patients exhibited significantly enhanced expression of c-Myc (*P* < 0.01) (Fig. 6a and b). Importantly, the high-level expression of c-Myc was positively correlated with the clinical stages of NPC patients (*P* = 0.014) (Table 1), indicating that c-Myc, as an oncogenic transcription factor, plays crucial roles in NPC progression.

Consistent with our previous data, the expression of BRD7 protein was significantly decreased (*P* < 0.001), and on the contrary, the level of miR-141 was overexpressed in NPC tissues compared with the normal controls (*P* < 0.001) (Fig. 6a and b). Considering the mechanism of BRD7 and c-Myc in miR-141 transcription, we further explored the association of c-Myc, BRD7 and miR-141 expression in NPC patients. The observations validated that the expression of c-Myc was significantly positively correlated with the level of miR-141 (*P* = 0.008) and negatively associated with the expression of BRD7 (*P* = 0.001) in NPC patients (Table 2). More importantly, we have previously demonstrated that BRD7 or miR-141 could serve as an independent prognostic factor of NPC [23]. In total, these results suggested that the feedback loop formed by BRD7 and c-Myc in miR-141 transcription is of great importance in NPC progression (Fig. 6c) and could serve as potential biomarkers for early detection and prognosis of NPC.

**Table 2:** Association of c-Myc, BRD7 and miR-141 expression in human NPC patients (*N* = 41)

	c-Myc expression		
	High	Low	*P*
miR-141 expression			0.0080
High	19	7	
Low	4	11	
	BRD7 expression		
	High	Low	*P*
miR-141 expression			0.0020
High	2	24	
Low	8	7	
	c-Myc expression		
	High	Low	*P*
BRD7 expression			0.0010
High	1	9	
Low	22	9	

## Discussion

MiR-141 is located on chromosome 12p13.31, and as a member of the miR-200 family, it is dysregulated in a wide variety of cancers [28, 29]. The genetic basis for aberrant miRNA expression is complicated in cancers. In FANTOM5 project, an integrated expression atlas of miRNAs and their promoters has been recently created and revealed that transcription factors are broadly involved in the regulation of specific miRNAs [30]. For example, TP53 regulated the miR-15/16 cluster [31], c-Myc modulated the miR-17-92 cluster [32], and HIF-1alpha targeted miR-210 [33]. In our previous study, we documented that BRD7 could negatively regulate miR-141 expression in an indirect manner at the transcription level [23]. Herein, we found that c-Myc knockdown decreased the levels of pri-miR-141 and pre-miR-141 in NPC cells, and downregulation was correlated with the expression of miR-141, which is consistent with our previous results derived from the differentially expressed miRNA profile in c-Myc knockdown NPC cells [12]. Moreover, we demonstrated that c-Myc bound directly to the miR-141 promoter and activated its transcription. In addition, c-Myc knockdown in NPC cells did not influence the expression of Dicer and Drosha proteins, two key enzymes that are indispensable to miRNA biogenesis and maturation. All of these observations together suggest that miR-141 is tightly regulated by transcription factors through a proximal promoter and that aberrant activation of c-Myc is at least partially responsible for the dysregulation of miR-141 in NPC.

c-Myc is a member of the proto-oncogenic bHLH transcription factor family. Numerous studies have identified that no monomeric c-Myc protein is present in vivo, and instead, c-Myc usually interacts with a partner protein, such as Max, Mad1, Mxi1 and Mnt, which is essential for the dimer to directly bind to the general E-box [26, 34, 35]. Indeed, c-Myc plays a dual role in the transcription activation or repression of its target genes, and this transition practically depends on the interaction partner of c-Myc [8]. For example, p19^ARF^ inhibits cell proliferation in a p53 stabilization-dependent manner and also functions in a p53-independent way by binding to c-Myc [36, 37]. BRD7, as a ubiquitous nuclear factor, is frequently involved in transcriptional regulation through protein-protein interactions with other transcription factors, such as TP53, BRCA1 and Smads [20, 22, 38]. In this study, we report that BRD7 colocalized with c-Myc in a punctate distribution pattern and interacted with c-Myc. Meanwhile, overexpression of BRD7 did not affect the mRNA level and protein stability of c-Myc. Importantly, we have previously performed an integrative analysis of genome-wide chromatin occupancy of BRD7 by ChIP-sequencing and digital gene expression profiling by RNA-sequencing and identified that there was no more than one binding-site of BRD7 within the miR-141 promoter region [27]. Interestingly, BRD7, as a direct target of c-Myc, could negatively regulate the transcription of miR-141 in an indirect manner in NPC [23, 24]. More importantly, we now document BRD7 being able to impair the transcriptional activation of miR-141 by c-Myc in NPC cells. Consequently, in light of the results observed above, it is apparent that the activity of miR-141 transcription was enhanced via BRD7 inactivation by c-Myc, and BRD7 could also serve as a cofactor of c-Myc to negatively regulate the activity of c-Myc/miR-141 transcription axis, suggesting that BRD7 and c-Myc might form a negative feedback loop responsible for the regulation of miR-141 expression.

Aberrantly elevated expression of c-Myc was highly prevalent in a variety of cancers and has been widely demonstrated as contributing to almost every aspect of tumor cell biology [39]. Several studies have shown that in c-Myc-driven malignancies, blocking continuous activation of c-Myc leads to growth inhibition and apoptosis [40]. Here, we confirm that knockdown of c-Myc causes NPC cell proliferation repression and tumor growth arrest. Additionally, the restoration of miR-141 can significantly rescue the tumor suppressive effect of c-Myc knockdown in NPC cells in vitro and in vivo. PTEN is a well-known lipid phosphatase and bona fide tumor suppressor, and numerous studies have identified a variety of cancers that arise following loss of PTEN function, which finally leads to a potent derepression of the PI3K/AKT pathway that stimulates cell proliferation and survival [41]. We have described previously that BRD7 has potent anti-proliferative and tumor suppressor activity at least partially through transcriptionally repressing miR-141, which directly targets the 3’UTR of PTEN mRNA and subsequently contributes to AKT pathway activation in NPC [12, 23]. Here, we document that knockdown of c-Myc activates PTEN/AKT signaling via transcriptional downregulation of miR-141 in NPC cells. Numerous observations indicated that following the downregulation of c-Myc, p27 is activated and cyclin D/CDK activity is repressed, resulting in cell proliferation inhibition by arresting G0/G1 phase progression [42, 43]. In accordance with previous identification, we validated that c-Myc knockdown induces G0/G1 phase arrest in NPC cells and that miR-141 can significantly reverse the tumor suppressive effects of c-Myc knockdown partially through restoring the expression of cyclin D1 and p27 in vitro and in vivo via the PTEN/AKT pathway. All of these results are consistent with the previous realization that minute variations of PTEN protein possess potent consequences for tumor initiation and cancer susceptibility [44] and suggest that the c-Myc/miR-141 axis regulates NPC growth at least partially through modulating the PTEN/AKT pathway.

We have previously reported that miR-141 was significantly upregulated while BRD7 was downregulated in NPC tissues, and both of them could serve as an independent prognostic factor of NPC patients [23]. In this work, we report that the expression of c-Myc is significantly upregulated in NPC patients, and the high-level expression of c-Myc is significantly correlated with advanced clinical stages of NPC. More importantly, the expression of c-Myc is positively associated with the level of miR-141 and negatively associated with the expression of BRD7 in NPC patients. Therefore, it is tempting to speculate that abnormalities in the feedback loop between BRD7 and c-Myc might be particularly required for NPC initiation and progression.

## Conclusion

Our findings demonstrated that c-Myc transactivates miR-141 expression and that BRD7, a direct target of c-Myc, serves as a cofactor of c-Myc and forms a negative feedback loop with c-Myc in miR-141 transcription in NPC. Moreover, the c-Myc/miR-141 axis promotes NPC growth through modulating the PTEN/AKT pathway. Furthermore, the feedback loop in miR-141 transcription is of great significance for early detection and prognosis of NPC. All of these observations provide new mechanistic insights into the dysregulation of miR-141 expression and a promising therapeutic option to interrupt the aberrant activation of miR-141 contributing to NPC progression.

## Additional files

Additional file 1: **Table S1.** The primers for confirmation of the c-Myc-binding sites in the miR-141 and BRD7 promoter. (DOC 34 kb)

Additional file 2: **Figure S1.** c-Myc did not influence the processing of pri-miR-141 and pre-miR-141 in miR-141 biosynthesis. **Figure S2.** BRD7 was inactivated by c-Myc and did not affect the stability of c-Myc protein. **Figure S3.** The levels of miR-141 restoration in c-Myc knockdown NPC cells. (DOC 2382 kb)

Additional file 3: **Table S2.** Ten putative c-Myc-binding sites were predicted in the miR-141 promoter region from the JASPAR Database. (DOC 40 kb)

Additional file 4: **Table S3.** Protein-Protein Interaction predictions for BRD7. (XLS 49 kb)

## Abbreviations

3’UTR: 3′ untranslated region
bHLH: Basic-helix–loop–helix
ChIP: Chromatin immunoprecipitation
Co-IP: Co-immunoprecipitation
FANTOM: Functional Annotation of Mammalian Genome
IF: Immunofluorescence
IHC: Immunohistochemical
ISH: In situ hybridization
miRNAs: microRNAs
NPC: Nasopharyngeal carcinoma
PPI: Protein-Protein Interaction
pre-miR-141: Precursor miR-141
pri-miR-141: Primary miR-141 transcript
qRT-PCR: Quantitative Real-Time polymerase chain reaction
shRNA: Short hairpin RNA
