# Supplementary material for: BRD7 expression and c-Myc activation forms a double-negative feedback loop that controls the cell proliferation and tumor growth of nasopharyngeal carcinoma by targeting oncogenic miR-141
Source: J Exp Clin Cancer Res. 2018 Mar 20;37:64. doi: 10.1186/s13046-018-0734-2 (PMC5859396; doi:10.1186/s13046-018-0734-2)
Supplement: Supplementary file 1 — Table S1. The primers for confirmation of the c-Myc-binding sites in the miR-141 and BRD7 promoter. (DOC 34 kb) [file 13046_2018_734_MOESM1_ESM.doc]

| **c-Myc-binding sites** | | **Primer pairs (5’-3’)** |
| --- | --- | --- |
| in the miR-141 promoter | | |
| R1 | Forward: ATTTGTCACCTGGTGGATCCAG | |
|  | Reverse: AGACCATCCAACACTGTACTGG | |
| R2 | Forward: AGAAGGAAGGAGGAAGAGCG | |
|  | Reverse: ACACACACCGATTTACCCAC | |
| R3 | Forward: TGAGACTAGGCAGGTTGGAG | |
|  | Reverse: TCTTCCTCCTTCCTTCTCCGC | |
| R4 | Forward: TCCCAGCACAGGCTGGGCAC | |
|  | Reverse: TCCAACCTGCCTAGTCTCACCCCTG | |
|  | | |
| in the BRD7 promoter | Forward: AGACGAGAGTCTGAGCGGTGG | |
|  | Reverse: ATGCCCCTCTCGAGAAGACGG | |

**Table S1.** The primers for confirmation of the c-Myc-binding sites in the miR-141 and BRD7 promoter
