# Supplementary material for: BRD7 expression and c-Myc activation forms a double-negative feedback loop that controls the cell proliferation and tumor growth of nasopharyngeal carcinoma by targeting oncogenic miR-141
Source: J Exp Clin Cancer Res. 2018 Mar 20;37:64. doi: 10.1186/s13046-018-0734-2 (PMC5859396; doi:10.1186/s13046-018-0734-2)
Supplement: Supplementary file 2 — Figure S1. c-Myc did not influence the processing of pri-miR-141 and pre-miR-141 in miR-141 biosynthesis. Figure S2. BRD7 was inactivated by c-Myc and did not affect the stability of c-Myc protein. Figure S3. The levels of miR-141 restoration in c-Myc knockdown NPC cells. (DOC 2382 kb) [file 13046_2018_734_MOESM2_ESM.doc]

**Figure S1**


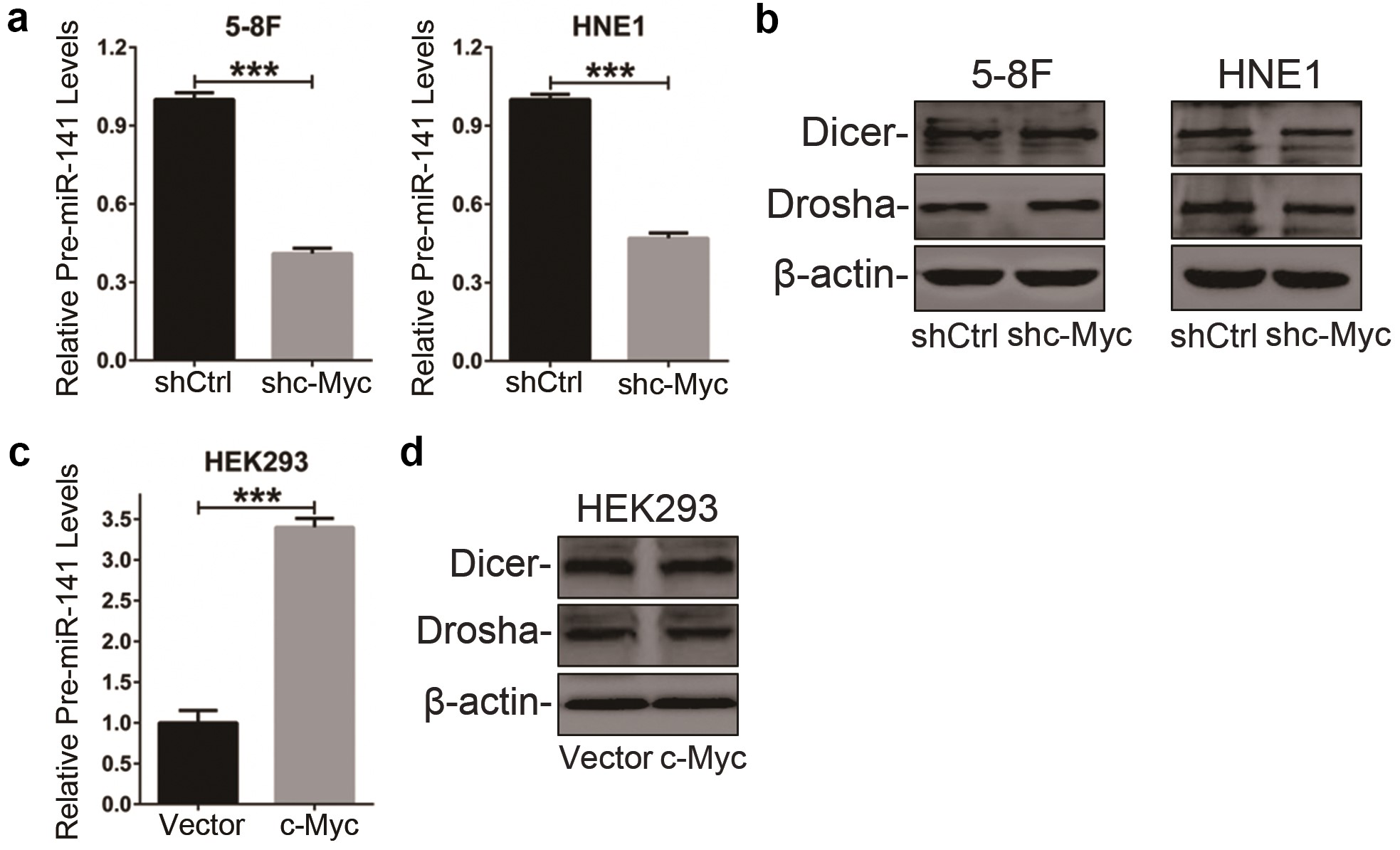


**Figure S1**. c-Myc did not influence the processing of pri-miR-141 and pre-miR-141 in miR-141 biosynthesis. **a** and **c** The level of pre-miR-141 was assessed by qRT-PCR in c-Myc knockdown stable 5-8F and HNE1 cells (shc-Myc) and control cells (shCtrl), and in c-Myc-overexpressing HEK293 cells (c-Myc) and control cells (Vector), respectively. **b** and **d** Western blotting determined the expression of Dicer and Drosha in c-Myc knockdown 5-8F and HNE1 cells, and in c-Myc-overexpressing HEK293 cells, respectively. **a** and **c** U6 served as an internal control. The error bars represent the mean±S.E.M. ***P<0.001. **b** and **d** β-actin served as an internal control.

**Figure S2**


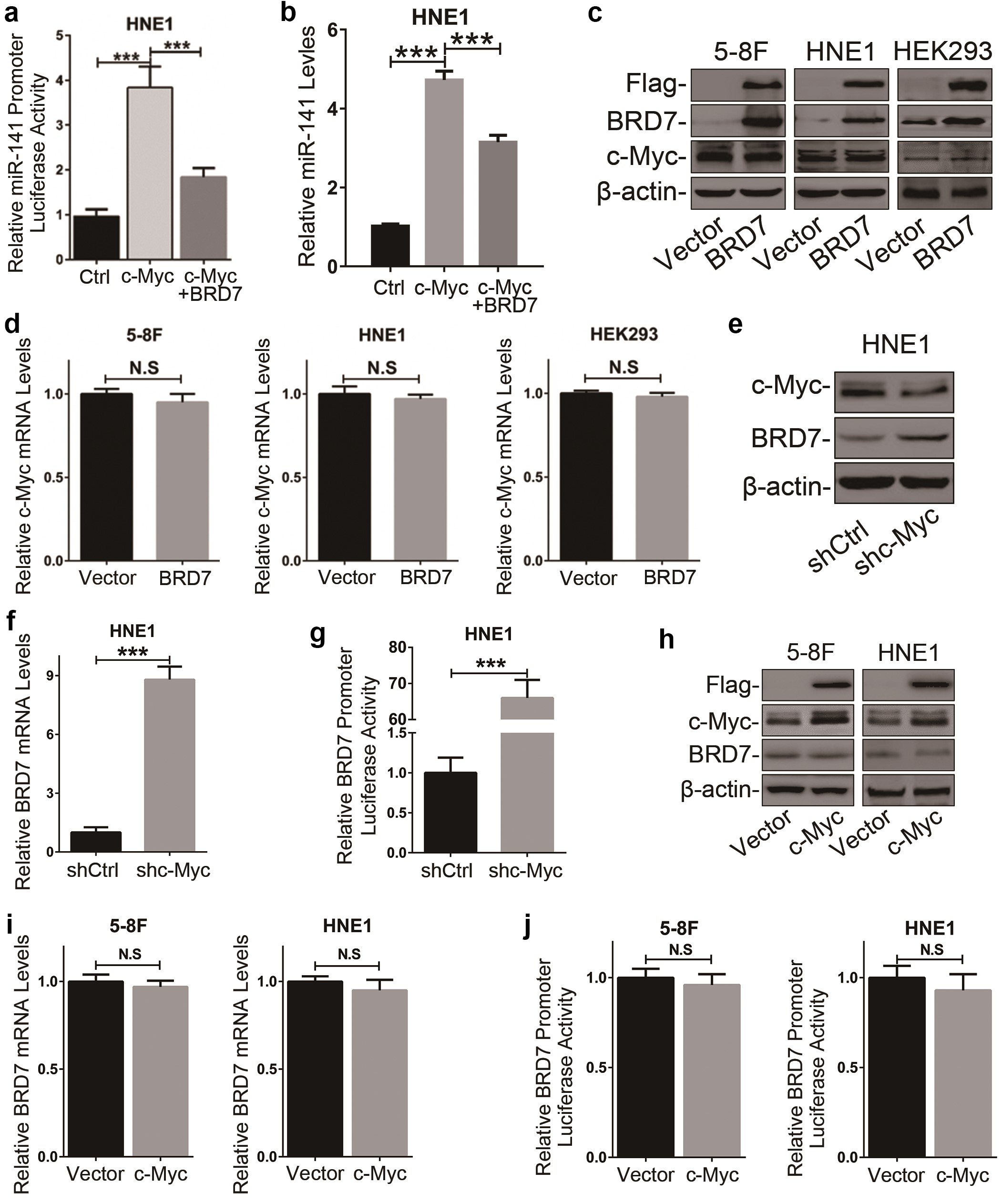


**Figure S2**. BRD7 was inactivated by c-Myc and did not affect the stability of c-Myc protein. **a** The dual-luciferase reporter assays assessed the miR-141 activity promoter when overexpressing BRD7 in c-Myc-overexpressing HNE1 cells (c-Myc) and control cells (Vector). **b** qRT-PCR assays evaluated the expression of miR-141 in c-Myc-overexpressing HNE1 cells when overexpressing BRD7. **c** Western blotting and **d** qRT-PCR assays evaluated the expression of c-Myc protein and mRNA, respectively, in BRD7-overexpressing 5-8F, HNE1 and HEK293 cells (BRD7) and control cells (Vector). **e** Western blotting and **f** qRT-PCR assays evaluated the expression of BRD7 protein and mRNA, respectively, in c-Myc knockdown HNE1 cells (shc-Myc) and control cells (shCtrl). **g** The dual-luciferase reporter assays determined the BRD7 promoter activity in c-Myc knockdown HNE1 cells and control cells. **h** Western blotting and **i** qRT-PCR assays analyzed the expression of BRD7 protein and mRNA in c-Myc-overexpressing 5-8F and HNE1 cells and controls. **j** The dual-luciferase reporter assays determined the BRD7 promoter activity in c-Myc-overexpressing 5-8F and HNE1 cells. **b**, **d**, **f** and **I** U6 or GAPDH served as an internal control. **c**, **e** and **h** β-actin served as an internal control. **a**, **b**, **d**, **f**, **g**, **i** and **j** The error bars represent the mean±S.E.M. ***P<0.001. N.S=No Significance.

**Figure S3**


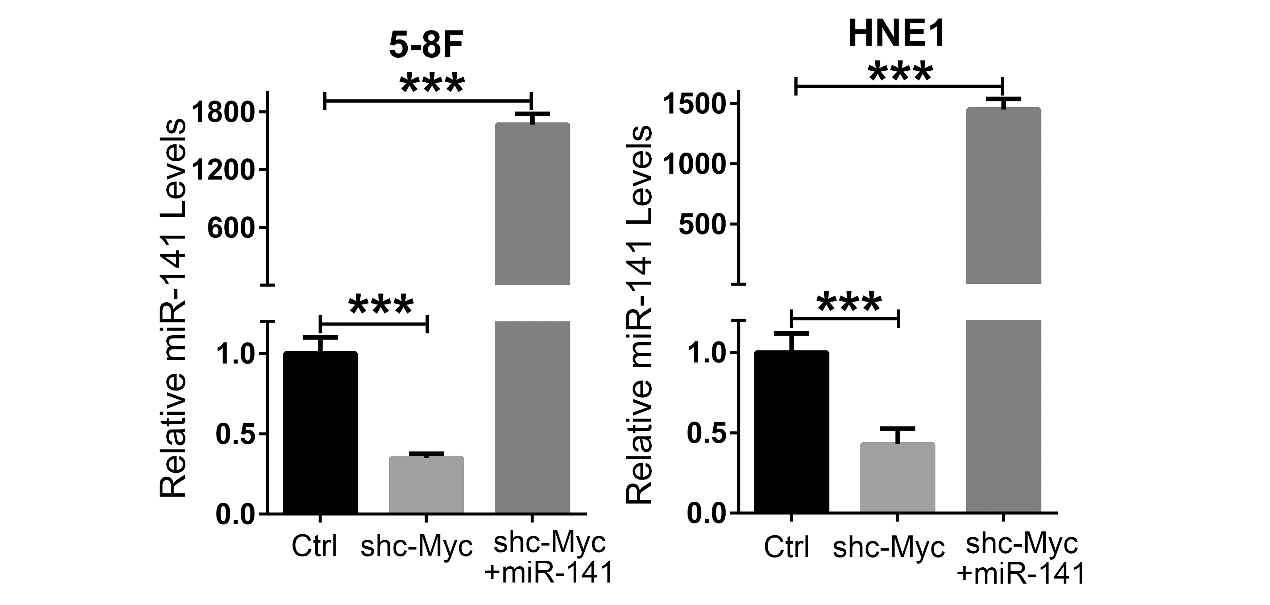


**Figure S3.** The levels of miR-141 restoration in c-Myc knockdown NPC cells. The level of miR-141 restoration was confirmed by qRT-PCR assays. U6 served as an internal control. Ctrl: shCtrl+miR-NC; shc-Myc: shc-Myc+miR-NC. The error bars represent the mean±S.E.M. ***P<0.001.
