# Supplementary material for: BRD7 expression and c-Myc activation forms a double-negative feedback loop that controls the cell proliferation and tumor growth of nasopharyngeal carcinoma by targeting oncogenic miR-141
Source: J Exp Clin Cancer Res. 2018 Mar 20;37:64. doi: 10.1186/s13046-018-0734-2 (PMC5859396; doi:10.1186/s13046-018-0734-2)
Supplement: Supplementary file 3 — Table S2. Ten putative c-Myc-binding sites were predicted in the miR-141 promoter region from the JASPAR Database. (DOC 40 kb) [file 13046_2018_734_MOESM3_ESM.doc]

**Table S2.** Ten putative c-Myc-binding sites were predicted in the miR-141 promoter region from the JASPAR Database

| **Model ID** | **Model name** | **Score** | **Start** | **End** | **Strand** | **Predicted site sequence** |
| --- | --- | --- | --- | --- | --- | --- |
| MA0147.2 | c-Myc | 3.305 | 48 | 57 | - | ACCCGTGCCC |
| MA0147.2 | c-Myc | 2.672 | 420 | 429 | - | GCCTGTGCTG |
| MA0147.2 | c-Myc | 2.672 | 562 | 571 | + | GCATCTGCTG |
| MA0147.2 | c-Myc | 4.567 | 703 | 712 | - | CCACGGGCAC |
| MA0147.2 | c-Myc | 2.417 | 705 | 714 | + | GCCCGTGGCT |
| MA0147.2 | c-Myc | 2.702 | 768 | 777 | - | ACAGGTGTGT |
| MA0147.2 | c-Myc | 2.764 | 772 | 781 | + | ACCTGTGCGC |
| MA0147.2 | c-Myc | 4.918 | 931 | 940 | - | CCACCTGCCC |
| MA0147.2 | c-Myc | 2.231 | 933 | 942 | + | GCAGGTGGGC |
| MA0147.2 | c-Myc | 2.368 | 1646 | 1655 | + | GCCTGTGGCC |
